# Background of New Measurement Electronic Devices with Polyelectrolyte Hydrogel Base

**DOI:** 10.3390/polym17040539

**Published:** 2025-02-19

**Authors:** Kaisarali Kadyrzhan, Ibragim Suleimenov, Lyazat Tolymbekova, Gaini Seitenova, Eldar Kopishev

**Affiliations:** 1 Department of Telecommunication Engineering, Institute of Communications and Space Engineering, Gumarbek Daukeev Almaty University of Power Engineering and Communications, Almaty 050040, Kazakhstan; kaisarali1997ss@gmail.com; 2 National Engineering Academy of the Republic of Kazakhstan, Almaty 050010, Kazakhstan; esenych@yandex.kz; 3 Department of Chemistry, Faculty of Natural Sciences, L.N. Gumilyov Eurasian National University, Astana 010000, Kazakhstan; tolymbekova_lb@enu.kz

**Keywords:** smart materials, electric field, polyelectrolyte hydrogels, water demineralization, electric double layer, induced EMF

## Abstract

It has been demonstrated that when a low-molecular-weight salt solution flows through a polyelectrolyte gel, an electromotive force is generated, and its polarity depends on the sign of the polyelectrolyte network’s charge. A mathematical model proving the possibility of developing a device for separating a solution of low-molecular salt into enriched and depleted phases under the influence of gravitational forces has been developed. Such a device contains a system of parallel columns filled with different kinds of cross-linked polyelectrolyte networks. The proposed mathematical model is grounded in the theory of double electrical layers forming at the hydrogel/solution interface; these layers deform under non-equilibrium conditions, specifically during the flow of the solution through the cross-linked polyelectrolyte network. An analogous model is proposed describing the case of an analogous device based on an electric current passing through two oppositely charged contacting networks, which provides the possibility of separating the initial solution into enriched and the depleted phases too. The practical applications of the found effect are discussed. In particular, it is demonstrated that a wide number of measurement electronic devices can be created on such a base, including devices to be used within the investigation of polyelectrolyte hydrogels of different types.

## 1. Introduction

Nowadays, various gel types find wide use in measurement technology: from gel permeation chromatography [1,2] to numerous examples of the application of hydrogels for the construction of biocompatible interfaces [3,4].

Such wide possibilities for the use of polyelectrolyte hydrogels for the development of a new generation of sensors and actuators with various applications are based on the pronounced sensitivity of polyelectrolyte hydrogels to many stimuli [5,6]. Thus, depending on temperature [7,8], ionic strength [9,10], the concentration of some specific substances [11,12,13], mechanic pressure [14,15], and electric current [16,17], polyelectrolyte hydrogels can change their volume in a reversible way. It is also significant to notice that recently, the development of the hydrogel-based analogues of electronic systems has also received extensive attention in the literature [18,19]. However, these achievements in no way exclude the possibility of alternative measuring devices that could exploit the specific features of polyelectrolyte hydrogels, which are capable of investigating the electrophysical properties of cross-linked networks that acquire an electrostatic charge due to the dissociation of functional groups.

Especially at the boundary between the solution of low-molecular-weight salt and the polyelectrolyte hydrogel, where double electric layers are formed. An example of a theory of their formation is given in [20]. In an equilibrium state, the appearance of such layers is closely related to the establishment of the Donnan equilibrium [21] between the gel volume and the surrounding solution. The mobility of the counterions within the gel volume allows for the coinciding of the salt concentrations inside and outside of the hydrogel, which may serve the development of the technologies of water demineralization [22,23]. Other cross-linked networks, namely ionites [24,25], possess the same property and find wide application in different membrane technologies [26,27], among which electrodialysis units are used in a variety of applications [28,29].

The measurement of the parameters of the aforementioned double layers, as well as the other electrophysical characteristics of cross-linked polyelectrolyte networks, provides valuable information about their properties, such as the degree of the ionization of the functional groups within the network. The diversity of the electrophysical characteristics of polyelectrolyte networks, in turn, raises the question of developing additional laboratory equipment specifically designed for their measurement.

This approach is further justified by the following considerations. Currently, the range of synthesized polyelectrolyte hydrogels [30,31,32,33], which were intended for use in a very wide and ever-increasing field of applications. Those materials have gained much interest due to their unique properties, including a high water-holding capacity, biodegradability, and a self-healing ability. All the medical applications of polyelectrolyte hydrogels include their use as drug delivery vehicles [34,35], biosensors [36,37], and material science smart coatings and the components responding to external stimuli such as pH, temperature, or ionic strength changes [38,39]. The fact that they can change in response to external stimulus shows that they might be applied in various fields from ecology and agriculture to electronics [40,41]. In the nearest future, a development of electrodialysis plants for all sorts of purposes is foreseen, and polyelectrolyte hydrogels are destined to play the role of highly efficient ion exchange membranes [42,43]. The foundation of such technologies is essentially based on the electrophoretic properties of cross-linked polymer networks.

This study demonstrates that when a low-molecular-weight salt solution flows through a cross-linked polyelectrolyte network, a difference in electrostatic potentials arises. This phenomenon is largely analogous to the streaming current and streaming potential effects [44,45]. The key distinction, however, is that in the present case, the effect is governed by processes occurring within a single phase, which can be considered homogeneous.

Additionally, this study proposes a theoretical framework for interpreting the observed phenomenon. It is also shown that this theory can serve as the foundation for a family of measuring instruments designed to investigate the electrophysical properties of various types of polymer networks, as well as for the development of new methods for the demineralization of low-molecular-weight salt solutions.

Section 2 examines the experiments that demonstrate the emergence of an electromotive force (EMF) when a low-molecular-weight salt solution flows through a cross-linked polyelectrolyte network.

Section 3 provides a theoretical interpretation of the observed phenomenon and considers theoretical models that prove the possibility of creating a wide range of devices (including measuring instruments) based on this principle.

In particular, the theoretical framework presented in this study serves as the basis for developing instruments capable of measuring the degree of ionization of functional groups in hydrogels. This task cannot be accomplished using conventional pH meters, as the introduction of an additional electrode into the sample volume leads to its destruction and compromises the integrity of the experiment.

Furthermore, the proposed theory provides the foundation for the development of experimental equipment designed for the direct investigation of electric double layers, which form, in particular, when polyelectrolyte networks carrying opposite electrostatic charges come into contact.

## 2. Material

The hydrogel used was synthesized by free-radical copolymerization of acrylic acid (AA) in aqueous solutions. The synthesis involved acrylic acid (Sigma-Aldrich, 99%) as the monomer, N,N′-methylenebisacrylamide (MBAA) (Sigma-Aldrich, 98%) as the cross-linker, and an initiator system consisting of ammonium persulfate (APS) (Sigma-Aldrich, 98%) and N,N,N′,N′-tetramethylethylenediamine (TEMED) (Sigma-Aldrich, 99%). This method is a standard approach and has been utilized in numerous studies, particularly in [46,47].

MBAA was added at a molar ratio of 300:1 (AA:MBAA) to ensure the formation of a stable polymer network. Polymerization was initiated by adding ammonium persulfate as an oxidizing agent along with TEMED as a co-initiator. The concentration ratio was chosen based on a compromise. A network that is too sparse would not allow for the formation of a pronounced potential difference, while a network that is too dense would prevent the achievement of a sufficiently high flow rate of the low-molecular-weight salt solution. The reaction mixture was then placed in a thermostat and maintained at 50 °C for 2–4 h to complete the polymerization and gelation processes. After polymerization, the hydrogel was partially neutralized with sodium hydroxide (NaOH) up to 75%, converting a portion of the acrylic acid units into sodium polyacrylate.

To remove residual impurities, the hydrogel was first dried using a fore-vacuum pump until a constant weight was achieved. After the initial drying, the sample was mechanically ground to obtain a uniform particle size. The hydrogel was then allowed to swell in pure water to remove any remaining unreacted components or by-products. After the swelling process, the hydrogel was once again dried to a constant weight using a fore-vacuum pump and subsequently ground again to ensure uniformity and complete purification.

## 3. Experiments

**Figure 1:**
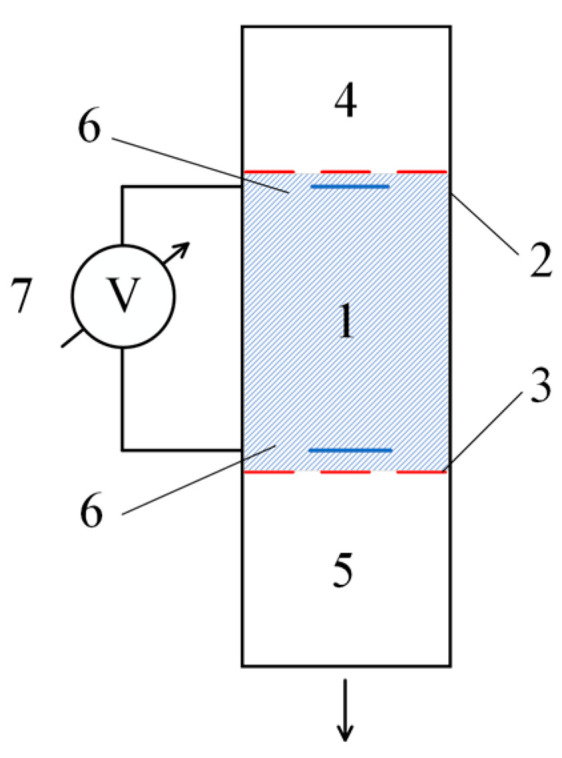
Experimental setup for detecting the flow potential of a solution flowing through the hydrogel. The numbers represent the following: 1—hydrogel-filled tube (length: 50 mm, diameter: 20 mm); 2, 3—membranes separating the hydrogel from the low-molecular-weight salt solutions; 4—volume filled with a low-molecular-weight salt solution (height: 5 mm, volume: 10 mL); 5—buffer volume collecting the solution that passes through the hydrogel layer (volume: 50 mL); 6—electrodes for measuring the dynamic potential difference (sensitivity: millivolt range); and 7—precision voltmeter capable of measuring small voltage differences (accuracy: ±0.01 mV).

The experimental setup is shown in Figure 1. It includes the following:

- A tube filled with hydrogel (1);
- Membranes (2) and (3), which separate the working material from the low-molecular-weight salt solutions used;
- A volume (4) filled with a low-molecular-weight salt solution;
- A buffer volume (5), which collects the low-molecular-weight salt that has passed through the hydrogel layer;
- Electrodes (6), intended for measuring the dynamic potential difference;
- A precision voltmeter (7), allowing measurements on the order of millivolts.

In the experiments, the gel (1) was placed between membranes (2) and (3). The volume (4) above the gel was filled with a low-molecular-weight salt solution, while the lower buffer volume (5) was initially filled either with deionized water or with a low-molecular-weight salt solution of the same concentration as in volume (4).

During the experiments, the difference in electrostatic potential arising from the flow of the salt solution through the gel was recorded. In parallel, the change in the height of the solution column above the gel and the potential difference were measured as functions of time.

The solutions used had the following characteristics. Ten milliliters of a 0.05 M KCl solution were poured into region (1). The buffer volume below the polyacrylic acid-based gel (a commercially produced hydrogel) was 50 mL. In the first series of experiments, the buffer was filled with deionized water.

Measurements were carried out without replacing the liquid (water), filling the buffer. Ten milliliters of the 0.05 M KCl solution were poured into a glass cylinder 20 mm in diameter, filled with the swollen PAA hydrogel to a height of 50 mm.

## 4. Results

**Figure 2:**
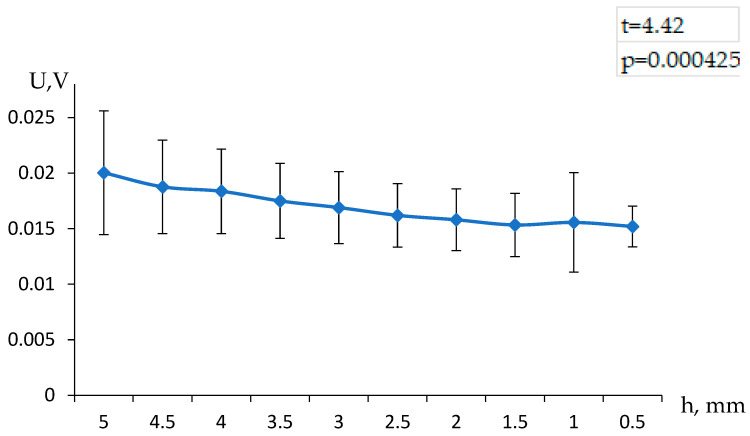
Dependence of the observed electric potential difference above and below the hydrogel on the height of the solution column above the hydrogel, when the buffer volume is filled with water.

An example of the result obtained for the five consecutive refills of the volume above the hydrogel is presented in Figure 2. It can be seen that the observed value of the potential difference depends on the height of the solution column above the gel, which directly indicates that the measured value depends on the flow rate of the solution.

To assess the statistical significance of the observed differences in electric potential above and below the hydrogel at varying solution column heights, a Student’s *t*-test was conducted. This test evaluates whether the differences between the two sets of data are statistically significant, determining if the variations are due to systematic factors rather than random fluctuations.

The conclusion drawn above is also confirmed by the experiments in which the liquid above and below the gel was a potassium chloride solution of the same concentration. In such a case, the appearance of a non-zero potential difference can only be attributed to the effects caused by the flow of the solution through the gel.

**Figure 3:**
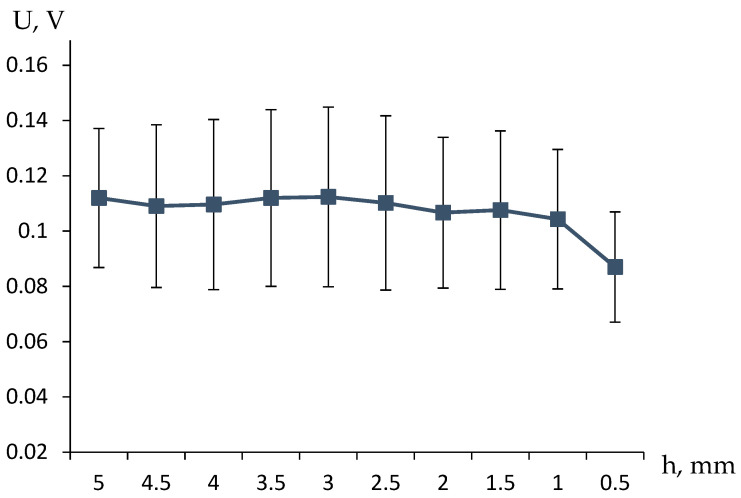
The dependencies of the observed electrical potential difference above and below the hydrogel on the height of the solution column above the gel, when the buffer volume is filled with a solution of the same concentration as above the gel, were obtained from three independent experimental runs, and the standard deviations were determined.

Experiments of this kind (examples of the results are presented in Figure 3) also showed a pronounced relationship between the measured voltage and the height of the solution column above the gel, and, consequently, the flow rate of the working fluid.

In our case, the *t*-test was applied to compare the voltage differences recorded in the experiments where the buffer volume was filled with water versus when it contained a solution of the same concentration as above the hydrogel. The resulting *t*-statistic and *p*-value indicate whether the observed differences are statistically significant.

The results of the *t*-test demonstrated a statistically significant difference between the two experimental conditions (*p* < 0.05), confirming that the solution flow through the hydrogel notably affects the recorded potential. This finding supports the hypothesis that the electric potential difference is influenced by the ionic movement within the hydrogel structure, rather than being a random effect. Such insights validate the reliability of using this method for further hydrogel-based electronic measurements and applications.

The experiment was conducted under the following conditions: the initial volume of the 0.05 M KCl solution poured above the gel was 10 mL, and the volume of the 0.05 M KCl solution below the hydrogel was 50 mL. A total of 10 mL of the 0.05 M KCl solution was added above the swollen (50 mm high) hydrogel in a 20 mm-diameter glass cylinder. The milled hydrogel was brought to an equilibrium degree of swelling before being placed in the cylinder.

The following section discusses a qualitative interpretation of the observed phenomenon. At the same time, it considers a setup that could potentially be used in the future to separate the low-molecular-weight salt flow into enriched and depleted streams.

## 5. Analysis

### 5.1. Qualitative Consideration of EMF Formation During the Flow of a Low-Molecular-Weight Salt Solution Through a Polyelectrolyte Hydrogel

**Figure 4:**
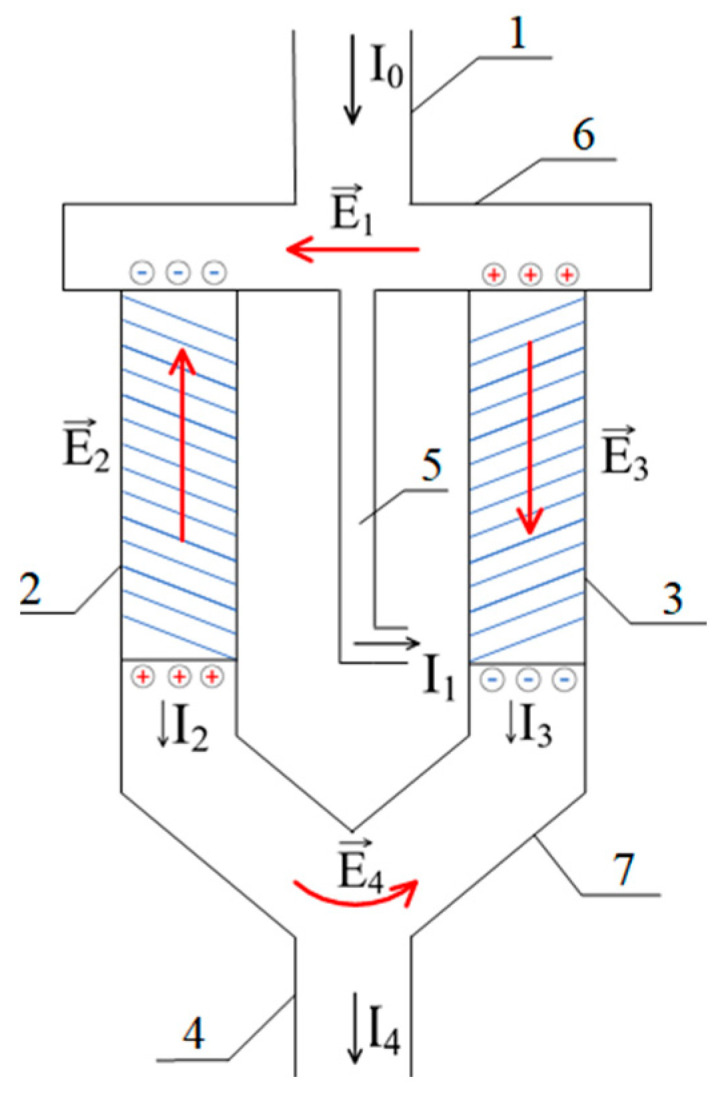
A variant of the diagram for separating the flow of low-molecular-weight salt into enriched and depleted. The numbers represent the following: 1—inlet for the feed solution of low-molecular-weight salt; 2, 3—column filled with a positively and negatively charged polyelectrolyte gel, respectively; 4—outlet for the depleted solution (salt concentration reduced); 5—outlet for the enriched solution (salt concentration increased); and 6, 7—auxiliary connecting elements facilitating the flow of the solution between the columns.

Figure 4 shows a variant of a scheme which is capable of separating a low-molecular-weight salt stream into an enriched and a depleted stream.

This scheme comprises the following:

- An outlet (1) through which the feed solution enters the system;
- A column (2) filled with a gel, the mesh of which carries a negative electrical charge;
- A column (3) filled with a gel whose mesh carries a positive electrical charge;
- Outlets for the depleted (4) and enriched (5) solutions;
- An auxiliary connecting elements (6) and (7).

In the schematic diagram shown in Figure 4, the initial solution flow enters columns 2 and 3, which are filled with positively and negatively charged hydrogels, respectively. Simultaneously, part of this flow is also discharged from the system through tube 5. The diameters of columns 2 and 3, as well as tube 5, are selected to ensure that the infiltration rate of the solution through the hydrogel-filled columns is comparable to the outflow rate through tube 5.

At the top, these three components of the system are connected by element 6. Similarly, the lower parts of columns 2 and 3 form a common outlet due to their connection via element 7. As demonstrated by the mathematical model presented below, this configuration can indeed lead to the separation of the solution into enriched and depleted streams due to the generation of an electromotive force (EMF) of the type investigated in this study.

The separation of the low-molecular-weight salt flow into enriched and depleted ones is achieved as a consequence of the formation of an electromagnetic field (EMF) within the system. Let us consider the nature of this process, beginning with the effect of concentration redistribution (in accordance with the terminology of [48]).

This effect can be attributed to the fact that the concentrations of the mobile oppositely charged particles inside the gel volume are not equal due to the neutrality condition. In particular, for a network carrying a negative charge (e.g., a network based on sodium polyacrylate) placed in a 1:1 salt solution, the next condition must be fulfilled:

(1) $$n_{g}^{+}=N_{0}+n_{g}^{-}$$

where $n_{g}^{\pm }$ are the concentrations of the ions of the corresponding sign, $N_{0}$ is the concentration of the dissociated functional groups of the network, and the index g denotes that the concentrations of ions in the hydrogel volume are considered.

In other notations, condition (1) is written as follows:

(2) $$C_{g}=N_{0}+c_{g}$$

where $C_{g}$ is the concentration of counterions, and $c_{g}$ is the concentration of low-molecular-weight salt inside the gel.

Equation (2) emphasizes that the concentration of low-molecular-weight salt inside the gel in the considered case coincides with the concentration of ions having the same charge sign as the polyelectrolyte mesh.

For the solution above the gel, the neutrality condition for the considered case of 1:1 salt is written in an obvious form:

(3) $$n_{s}^{+}=n_{s}^{-}=c_{s}$$

where the index s marks the concentrations in the solution above the gel, respectively, and $c_{s}$ is the salt concentration in this solution.

Then, supplement equations (1) and (3) with an equation linking the concentrations of low-molecular-weight ions outside and inside the hydrogel

(4) $$n_{g}^{\pm }=n_{s}^{\pm }\exp\mp \frac{e∆\varphi }{kT}$$

If one assumes that ∆φ is the electrostatic potential difference between the gel and the surrounding solution, *e* is the elementary charge, k is the Boltzmann constant, and T is the absolute temperature, it is straightforward to derive an expression that relates $c_{s}$ and $c_{g}$. This will show that the concentration of a low-molecular-weight salt inside the volume of a polyelectrolyte gel will always be lower than its concentration in the solution above the gel.

**Figure 5:**
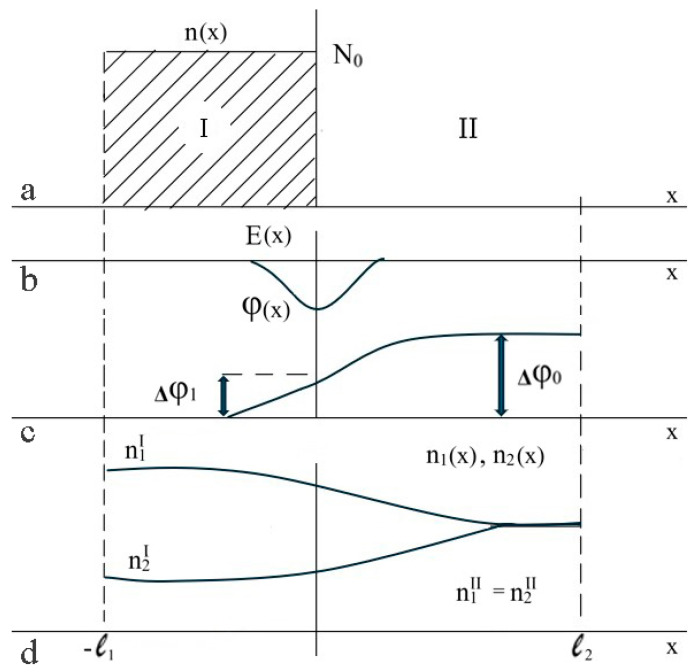
Illustration of the effect of the redistribution of concentrations: (**a**)—dependence of the concentration of ionized functional groups, n, on the coordinate, (**b**)—dependence of the electric field magnitude E of the double electric layer on the coordinate, (**c**)—dependence of the electrostatic potential φ on the coordinate, and (**d**)—dependence of the concentrations of the low-molecular ions of opposite signs n1 and n2 on the coordinate, the lower curve also corresponds to the behavior of the concentration of low-molecular salt [48].

This implies that the electrostatic potential difference, ∆φ, cannot be precisely zero (which is associated with the attainment of the Donnan equilibrium), since the change in the electrostatic potential takes place in a relatively limited region close to the hydrogel boundary. This is schematically illustrated in Figure 5c, which illustrates the dependence of the concentration of ionized hydrogel functional groups on the coordinate, in the case where the boundary of a flat sample is considered [48]. Consequently, at this point, an electrostatic field of non-zero magnitude is generated, the value of which is comparable to the Debye length (refer to Figure 5b). The dependence of the concentrations of low-molecular-weight ions on the coordinate is schematically shown in Figure 5d.

It is reasonable to assume that a continuous flow of fluid with low-molecular-weight ions will pass through the polyelectrolyte hydrogel. This will involve the transport of the mobile, charged particles created by the dissociation of functional groups (–COONa) in the network (counterions).

The transport of ions, together with fluid flow, will tend to violate the electrical neutrality condition in the medium inside the network. This follows from the fact that according to condition (2), the amplitudes of ion fluxes bearing opposite charges do not occur at the same time. The condition of neutrality can be maintained only when an electric field is generated in the network, which inhibits the movement of excess ions. It becomes obvious that the same electric field will accelerate the missing ions. The formation of this type of field results in an equalization of the currents of differently charged particles in cases when their concentrations are not equal.

The underlying physical mechanism that gives rise to the emergence of this field is related to the distortion of the double electric layers that form at the interface between the polyelectrolyte network and the surrounding solution. Such a process occurs when there exists a non-zero flow rate of the solution passing through the network. This claim is founded on the most general theoretical considerations. Non-zero electrostatic fields arise in these systems, even at equilibrium (see Figure 5b of reference [48]). To maintain symmetry, it is necessary to establish the form of the fields present in a flat gel sample equilibrated with the surrounding solution. The fluid flow in the gel disrupts this symmetry. Also, considering that departures from electrical neutrality can occur only at distances on the order of the Debye length, in non-equilibrium situations, the distribution of the concentrations of mobile ions, as well as those inside the gel and in the solution overlying the gel, is presumed to be homogeneous. One may conclude that the formation of the EMF, which guarantees equality of the fluxes of the low-molecular-weight ions within the gel, is mainly related to the deformation of the double electric layers.

The behavior in this case can be likened to that of a low-molecular-weight salt passing through two columns filled with polyelectrolyte networks bearing opposite charges. In order to maintain electrical neutrality, an electrostatic field is set up inside one of the networks that retards the anions, while inside the other network it retards the cations. It can thus be concluded that the above-mentioned domain is associated with an excess of electrostatic charge, which forms at the hydrogel/solution interface due to the distortion of the double electric layers. The presented idea is illustrated further by the schematic representation of charges shown in Figure 4. The $E_{2}$ electric field ensures the equality of amplitudes for the positive and negative ion fluxes in the left compartment, while ensuring equality in the right compartment is conducted by the $E_{3}$ electric field. The directions of the vectors representing these fields are opposite, resulting in the appearance of fields $E_{1}$ and $E_{4}$ and thus setting up the electrical circuit.

The currents generated by the $E_{2}$ and $E_{3}$ fields flow in opposite directions. However, in application practice, these currents support the increase in ion movements that carry the same charges through the respective polyelectrolyte matrix. The positively charged ions are driven through one kind of hydrogel while the negatively charged ions are driven through the other kind of hydrogel. The net effect of the fields changing along each column of the system is to augment the migration of both positively and negatively charged ions. Consequently, the rate at which low-molecular-weight salt crosses the membrane will be greater than the rate at which liquid flows across it. Turning back to the diagram shown in Figure 4, it is clear that the flux rate of low-molecular-weight salt through the outlet (5) will be equal to the flux rate of the solution. On the other hand, the flux rate of the low-molecular-weight salt through the outlet (4) will be greater than that of the liquid through the gel. Thus, if the geometry of the system is chosen correctly, the enriched solution will exit through outlet (4), and the depleted solution will exit through outlet (5). The proper justification of this conclusion is given in the following section, which is based on the mathematical model. It turns out that the mathematical model describing the enrichment and depletion of the flow of the solution in such a system can be derived based on an analysis of the current balance, which eliminates the necessity to conduct a comprehensive analysis of the properties of the deformations in the electrical double layers.

It should be noted that a similar methodology was already used in [20], in which it was demonstrated that the essential characteristics of the double electric layer formed at the interface between the hydrogel and solution can be deduced from the diagram shown in Figure 5.

### 5.2. EMF Formation During Salt Solution Flows Through a Polyelectrolyte Network

Let us first consider the simplest possible case when a 1:1 solution of a low-molecular-weight salt flows through a single column filled with a swollen polyelectrolyte gel, the mesh of which carries a negative charge, at a velocity *v*. This situation corresponds to the experiments discussed in Section 2. It follows from the neutrality condition that the fluxes of the ions of opposite charge signs $j_{2}^{+}$ and $j_{2}^{-}$ should be equal:

(5) $$j_{2}^{+}=j_{2}^{-}$$

Index 2 in this equation refers to the left column in Figure 4, which is filled with a polyelectrolyte mesh carrying a negative charge.

Each of these flows is formed due to two factors: the effect of the electric field and the transport of ions together with the fluid current (in the region where the field is homogeneous it is acceptable to neglect the contribution of the diffusion component).

(6) $$j_{2}^{+}=v_{2}n_{2}^{+}-q_{0}b^{+}E_{2}n_{2}^{+}$$

(7) $$j_{2}^{-}=v_{2}n_{2}^{-}+q_{0}b^{-}E_{2}n_{2}^{-}$$

where $n_{2}^{\pm }$ are the concentrations of positively and negatively charged ions in the considered column, $b^{\pm }$ are the mobility coefficients of the ions, $E_{2}$ is the electric field strength, and $q_{0}$ is the elementary charge.

By solving these equations for $E_{2}$, we obtain the following:

(8) $$q_{0}E_{2}=v_{2}\frac{n_{2}^{+}-n_{2}^{-}}{b^{+}n_{2}^{+}+b^{-}n_{2}^{-}}$$

Equation (8) gives the nonvanishing electric field in the system as solely arising due to the excess concentration of positively charged counterions, $n_{2}^{+}$, over the concentration of negative ions, $n_{2}^{-}$, present in one of the columns saturated with a negatively charged polyelectrolyte network. The compensatory mechanism is provided by the electric field whose strength is precisely proportional to the solution flow velocity through the grid.

The corresponding equations can be written for the right column (Figure 4) when filled with a positively charged polyelectrolyte network.

(9) $$j_{3}^{+}=v_{3}n_{2}^{+}+q_{0}b^{+}E_{3}n_{3}^{+}$$

(10) $$j_{3}^{-}=v_{3}n_{3}^{-}-q_{0}b^{-}E_{3}n_{3}^{-}$$

whence

(11) $$q_{0}E_{3}=v_{3}\frac{n_{3}^{-}-n_{3}^{+}}{b^{+}n_{3}^{+}+b^{-}n_{3}^{-}}$$

As noted above, the formation of the electrostatic field in the considered system is associated with the deformation of the double electric layers at the boundary of the gel and the solution. These layers cease to be symmetric, i.e., a layer of uncompensated electric discharge is formed at the mentioned boundaries (Figure 4).

**Figure 6:**
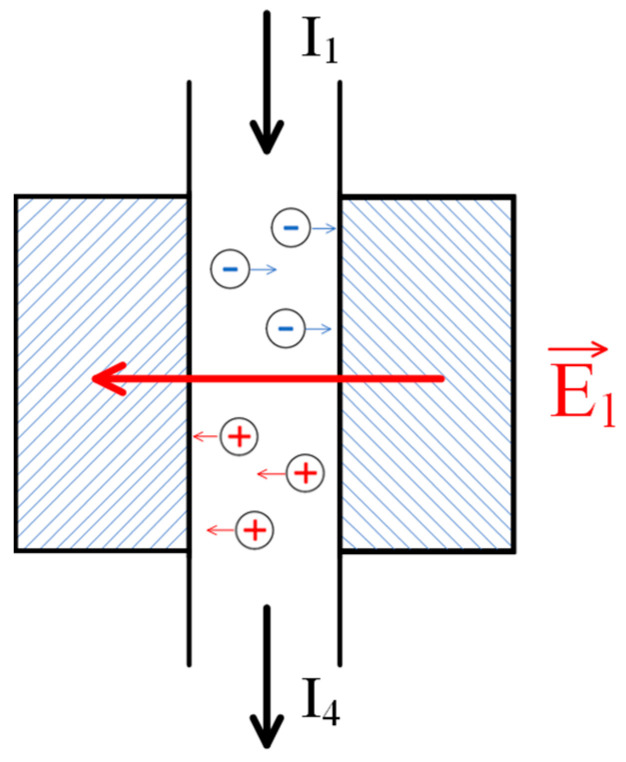
Formation of the analogue of the electrodialysis chamber in the considered system: $E_{1}$—the field generated due to the emergence of an electromotive force (EMF) caused by the flow of the solution through the polyelectrolyte networks; $I_{1}$—the flux of the initial solution; and $I_{4}$—the flux of the depleted solution.

Consequently, along with the fields $E_{2}$ and $E_{3}$, formed by the above mechanism, the fields $E_{1}$ and $E_{4}$ directed as shown in Figure 4, also appear in the system under consideration. As a result, the tube (6) in Figure 4 becomes an analogue of a separate section of a classical dialyzer. For clarity, the corresponding part of the circuit is separately shown in Figure 6. In it, the left column plays the role of an anion-exchange membrane, and the right column plays the role of a cation-exchange membrane. As in the usual scheme of a dialyzer [49,50], the electric field applied perpendicularly to the solution flow leads to its depletion, as the ions of opposite signs of charge are removed from the flow through the columns filled with the polyelectrolyte gel, which play the role of membranes.

As a result, non-zero flows of oppositely contaminated ions, which are not related to the movement of the solution as a whole, arise in the system under consideration. These flows, as in the dialyzer, lead to a depletion of the solution, which is discharged through channel (1), as shown in Figure 4. It can be written as follows:

(12) $$j_{2}^{+}=j_{2}^{-}+∆j$$

(13) $$j_{3}^{-}=j_{3}^{+}+∆j$$

The occurrence of additional flows leads to a change in the balance resulting in non-zero fields in the right and left columns, as shown in Figure 4. It is easy to show the following:

(14) $$q_{0}E_{2}=\frac{v_{2}\left(n_{2}^{+}-n_{2}^{-}\right)-∆j}{b^{+}n_{2}^{+}+b^{-}n_{2}^{-}}$$

(15) $$q_{0}E_{3}=\frac{v_{3}\left(n_{3}^{-}-n_{3}^{+}\right)-∆j}{b^{+}n_{3}^{+}+b^{-}n_{3}^{-}}$$

From these equations, in particular, it follows that a kind of equilibrium must be established in the system under consideration. An effect similar to that underlying electrodialysis leads to the appearance of additional fluxes of low-molecular-weight ions, which, in turn, lead to a decrease in the amplitude of the fields $E_{2}$ and $E_{3}$, causing the appearance of these fluxes. The same equation shows that there is indeed an enrichment of the flux of low-molecular-weight ions $I_{4}$ by the value ∆j, respectively, the flux $I_{1}$ is depleted by the same value.

### 5.3. Separation of Solution Streams into Enriched and Depleted by the Contact of Oppositely Charged Meshes

The analysis of the scheme of Figure 4 shows that the key role in the considered process is played by the electric fields arising without the use of an external current or voltage sources. The emergence of these fields, in turn, is associated with the balance of ionic currents in the grids carrying electric charges of different signs. A similar effect, theoretically, can occur when such meshes are in direct contact.

**Figure 7:**
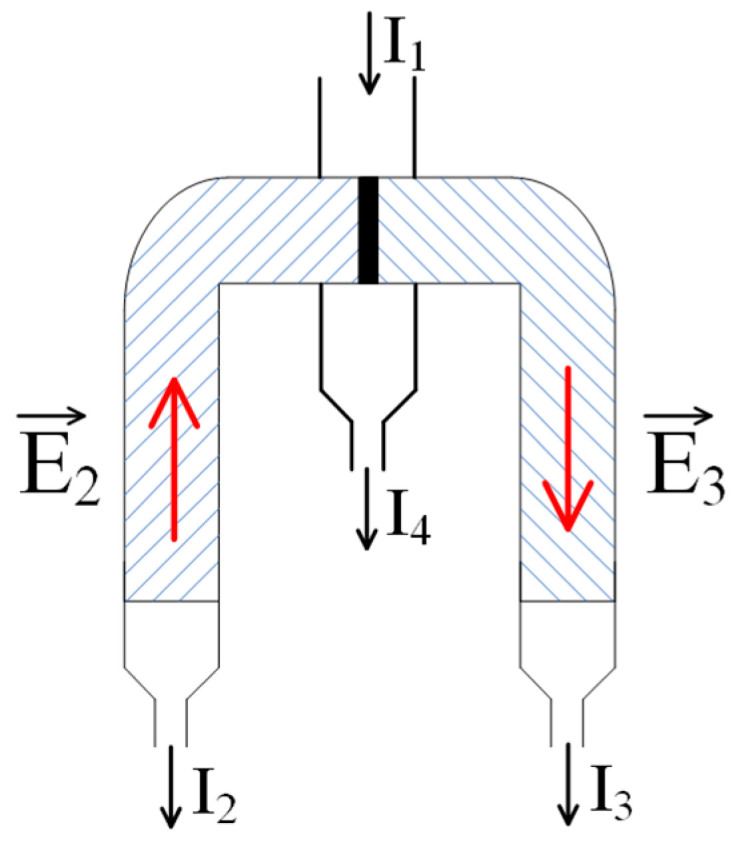
A modification of the scheme of Figure 4: a transition to the direct contact of oppositely charged meshes. $E_{2,3}$—fields generated due to the emergence of an electromotive force (EMF) caused by the flow of the solution through polyelectrolyte networks; $I_{1}$—inflow of the initial solution; $I_{4}$—outflow of the depleted solution; and $I_{2,3}$—flows of the solution enriched with oppositely charged ions.

Figure 7 shows a scheme similar to that of Figure 4, differing in that in it the networks carrying opposite charges are in direct contact. Again, it is assumed that the initial flow of the low-molecular-weight salt solution is split into three, with flows *I*_2_ and *I*_3_ being diverted through the columns filled with oppositely charged meshes.

**Figure 8:**
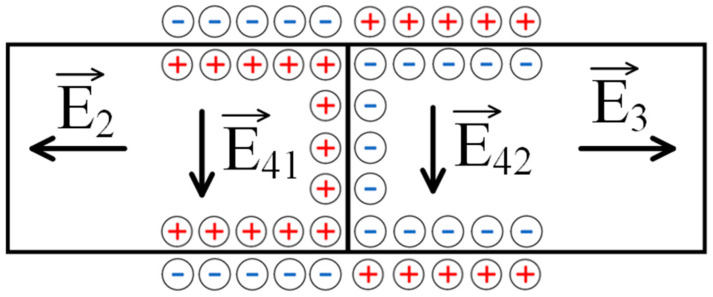
The mechanism of formation of electric fields in the context of the scheme presented in Figure 7: E_2,3_—fields arising due to the generation of an EMF caused by the flow of the solution through the polyelectrolyte networks; and E_41,42_—fields generated as a result of the solution flowing through the contact zone of oppositely charged networks.

**Figure 9:**
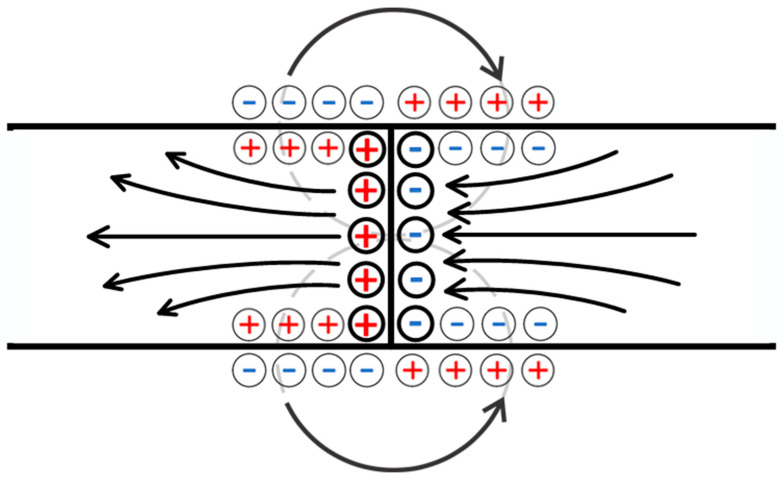
The anticipated configuration of the field within the contact zone of two oppositely charged networks. Bold circles highlight the schematic charges corresponding to the electric double layer formed through the contact of oppositely charged polyelectrolyte networks, while thin circles schematically indicate the charges corresponding to the double layers formed at the interface between the network and the solution.

Based on Equations (14) and (15), we can conclude that when a low-molecular-weight salt solution flows through the contact region of two oppositely charged networks, a very complex electric field structure is formed in it. Indeed, along with the fields $E_{2}$ and $E_{3}$ formed by the mechanism discussed above, fields of similar nature should arise directly in the contact region. The electric field vectors corresponding to the fluxes $I_{2}$ and $I_{3}$ on the one hand, and to the flux $I_{4}$ on the other hand (the latter flux consists of two) are schematically represented in Figure 8. The occurrence of the combination of fluxes, therefore, does lead to a complex field structure, which is schematically shown in Figure 9. This figure emphasizes that the field lines in the contact zone, among others, can extend beyond the charged networks, i.e., the resulting field system can, among others, influence the solution above the system under consideration.

This field structure can also provide a separation of the low-molecular-weight salt flow into enriched and depleted streams, since the scheme of Figure 7 is an analogue of the scheme of Figure 4. However, if one considers a scheme in which contacting oppositely charged networks is used, it makes sense to consider also a modification of the scheme of Figure 4 in which an external voltage source is used. Indeed, as follows from Figure 9, an electric field develops near the contact zone, which is able to stimulate the penetration of the low-molecular-weight ions into the polyelectrolyte networks. The strength of this field obviously depends on the amplitude of the double layer field formed in the contact zone of oppositely contaminated meshes. This amplitude can be increased by using a third-party current source.

On this basis, let us consider a system consisting of two oppositely charged networks through which an electric current from an external voltage source is passing.

The object arising from the contact between the two oppositely charged networks is, in many respects, analogous to a semiconductor diode, in which a double electric layer is formed at the boundary of the regions of electron and hole conductivity. This analogy, however, remains rather distant, since hole conduction in semiconductors is de facto associated with an electronic current. In the considered case, charge carriers of opposite signs are physically present in the system. Note that systems containing various types of charge carriers are currently being actively studied, including from the standpoint of developing neuromorphic materials [51,52,53].

The system of equations describing the charge distribution in each of the considered networks has the following form:

(16) $$-D^{+}\frac{d}{dx^{2}}n_{i}^{+}+q_{0}\frac{d}{dx}\left(b^{+}E_{i}n_{i}^{+}\right)=0$$

(17) $$-D^{-}\frac{d}{dx^{2}}n_{i}^{-}-q_{0}\frac{d}{dx}\left(b^{-}E_{i}n_{i}^{-}\right)=0$$

(18) $$\frac{dE_{i}}{dx}=\frac{q_{0}}{\varepsilon_{0}\varepsilon }\left(n_{i}^{+}-n_{i}^{-}\pm N_{0i}\right)$$

where $D^{\pm }$ and $b^{\pm }$ are the diffusion and mobility coefficients of the ions of the corresponding sign, $\varepsilon_{0}$ is the dielectric permittivity of the vacuum (Equations are written in SI system), and ε is the dielectric permittivity of the medium.

Equation (18) in this system follows from the classical equations of electrostatics; the only nuance is related to the consideration of the electrostatic charge carried by the polyelectrolyte network. The plus sign in this equation corresponds to a positively charged network, while the minus sign corresponds to a negatively charged network. To build an approximate mathematical model, it is also acceptable to use the well-known equation reflecting the relationship between the diffusion and mobility coefficients.

(19) $$D^{\pm }=b^{\pm }kT$$

Using Equation (19), the system of Equations (16)–(18) is reduced to the following form.

(20) $$-\frac{d}{dx}n_{i}^{+}+\frac{q_{0}}{kT}E_{i}n_{i}^{+}=\frac{j_{i}^{+}}{D^{+}}$$

(21) $$-\frac{d}{dx}n_{i}^{-}-\frac{q_{0}}{kT}E_{i}n_{i}^{-}=\frac{j_{i}^{-}}{D^{-}}$$

(22) $$\frac{q_{0}}{kT}\frac{dE_{i}}{dx}=\frac{q_{0}^{2}N_{0i}}{\varepsilon_{0}\varepsilon kT}\left(\frac{n_{i}^{+}-n_{i}^{-}}{N_{0i}}\pm 1\right)$$

It can be seen that in this system of equations there appears a parameter λ representing the Debye length [54] for the case when the integral ion concentration in the system is equal to $N_{0i}$, i.e.,

(23) $$\lambda^{2}=\frac{\varepsilon_{0}\varepsilon kT}{q_{0}^{2}N_{0i}}$$

Let us pass to the dimensionless coordinate, counted in units of λ,

(24) $$X=\frac{x}{\lambda },$$

as well as to the dimensionless electric field strength and concentration

(25) $$e_{i}=\lambda \frac{q_{0}}{kT}E_{i};\;N_{i}^{\pm }=\frac{n_{i}^{\pm }}{N_{0}}$$

This allows us to bring the system of equations under consideration into a dimensionless form. We have the following:

(26) $$-\frac{d}{dX}N_{i}^{+}+e_{i}N_{i}^{+}=\lambda \frac{j_{i}^{+}}{N_{0}D^{+}}=w_{i}^{+}$$

(27) $$-\frac{d}{dX}N_{i}^{-}-e_{i}N_{i}^{-}=\lambda \frac{j_{i}^{-}}{N_{0}D^{-}}=w_{i}^{-}$$

(28) $$\frac{de_{i}}{dX}=\left(N_{i}^{+}-N_{i}^{-}\pm 1\right)$$

To obtain conclusions at the qualitative level, which is the purpose of this study, there is no need to build a precision mathematical model. Consequently, at this stage of research, Equations (26)–(28) can be linearized, assuming that

(29) $$N_{i}^{\pm }=N_{i0}^{\pm }+\delta N_{i}^{\pm }$$

(30) $$e_{i}=e_{i0}+\delta e_{i}$$

The following relations must be fulfilled for the unperturbed values of the considered quantities.

(31) $$e_{i0}N_{i0}^{+}=w_{i}^{+}$$

(32) $$e_{i0}N_{i0}^{-}=w_{i}^{-}$$

(33) $$N_{i0}^{+}-N_{i0}^{-}\pm 1=0$$

Relations (31) and (32) actually express the balance of the low-molecular-weight ion fluxes in the system under consideration, and Equation (33) expresses the condition of electrical neutrality in the dimensionless form. Passing to the sum and difference of mobile ion concentrations, we obtain

(34) $$-\frac{d}{dX^{2}}\delta e_{i}+\delta e_{i}\left(N_{i0}^{+}+N_{i0}^{-}\right)+e_{i0}\left(\delta N_{i}^{+}+\delta N_{i}^{-}\right)=0$$

(35) $$\frac{d}{dX}\left(\delta N_{i}^{+}+\delta N_{i}^{-}\right)=\mp \delta e_{i}+e_{i0}\frac{d\delta e_{i}}{dX}$$

The system of Equations (34) and (35) is reduced to a single third order linear differential equation on the variation of the electric field.

(36) $$-\frac{d}{dX^{3}}\delta e_{i}+\beta \frac{d\delta e_{i}}{dX}\mp e_{i0}\delta e_{i}=0$$

where

(37) $$\beta =N_{i0}^{+}+N_{i0}^{-}+e_{i0}^{2}$$

The standard technique for solving linear differential equations with constant coefficients is based on a substitution of the form

(38) $$\delta e_{i}=\delta e_{i0}\exp\mu X$$

which leads to the following characteristic equation for the parameter μ

(39) $$-\mu^{3}+\beta \mu \mp e_{i0}=0$$

The characteristic scales of the parameters appearing in Equation (38) can be established from obvious considerations. If a network carrying a sufficiently appreciable electric charge is considered, the effect of the redistribution of concentrations is pronounced and, consequently, β=o(1). The characteristic value of the dimensionless electric field strength is determined by the relation

(40) $$e_{i}\sim \frac{\lambda }{\Lambda }$$

where

(41) $$\Lambda =\frac{kT}{q_{0}E_{m}}$$

Numerical estimates show that the length λ for systems of the type under consideration, as one would expect, does not exceed 10 nm, whereas at an electric field strength of 1 V/m the length Λ is 2.5 cm (2.5 mm for a field strength of 10 V/m). Consequently, Equation (38) is also acceptable to be solved by the perturbation method, considering the dimensionless parameter $\frac{\lambda }{\Lambda }$ to be small. Assuming

(42) $$\mu_{0}=\pm \sqrt{\beta }$$

and using the equation

(43) $$\mu =\pm \sqrt{\beta }+\delta \mu$$

we obtain

(44) $$\delta \mu =\mp \frac{e_{i0}}{3\sqrt{\beta }}$$

Equations (43) and (44) correspond to two solutions of the linear Equation (38). Along with them there is a third solution corresponding to small values of μ, namely

(45) $$\mu =\mp \frac{e_{i0}}{\beta }\sim \mp \frac{\lambda }{\Lambda }$$

The minus sign in this equation corresponds to the grid carrying a positive charge, and the plus sign corresponds to the negative one. We also emphasize that the exponent μ was calculated for the dimensionless coordinate (24), i.e., the characteristic scale for this solution is determined by the length Λ, which exceeds the Debye length by several orders of magnitude.

Thus, it can be seen that the behavior of the system under consideration is determined by a significant difference in the characteristic lengths Λ and λ, which reach several orders of magnitude.

Consequently, if we talk about the solutions corresponding to Equation (43), the influence of the electric field (δμ) can be neglected. This means that the character of the formation of the electric double layers associated with diffusion remains unchanged at those electric field values, which are reasonable to use in practice. Of much more interest is the third solution corresponding to exponent (44), the analogue of which is absent when the linearized solution of the Poisson–Boltzmann equation [55] is considered. Recall that when this equation is written for the case when the characteristics of the system depend only on one coordinate, it transforms into a second-order differential equation, which has two solutions. The third solution appears only when the medium inside the grid carrying a non-zero electric charge is considered.

**Figure 10:**
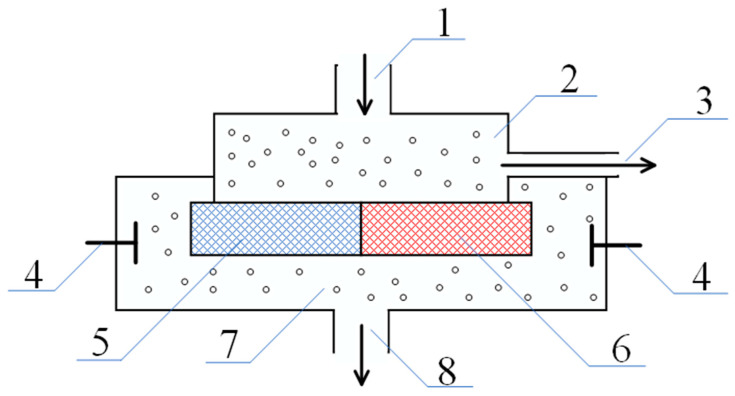
Modernized version of the scheme for the separation of the low-molecular-weight salt flow into enriched and depleted: 1—outlet for inflow of the initial solution of low-molecular salt into the system, 2—vessel filled with initial solution, 3—outlet for removal of depleted solution, 4—electrodes, 5—negatively charged network, 6—positively charged network, 7—vessel filled with depleted solution, and 8—outlet for depleted solution.

Starting with solution (45), the following scheme can be proposed to ensure the demineralization of water (Figure 10).

In this scheme, the network carrying the negative charge is located on the left, and the positive one on the right. Accordingly, for the left network the solution of the linearized equation has the form

(46) $$\delta e_{i}=\delta e_{i0}\exp\frac{x}{\beta \Lambda }$$

and for the right

(47) $$\delta e_{i}=\delta e_{i0}\exp-\frac{x}{\beta \Lambda }$$

**Figure 11:**
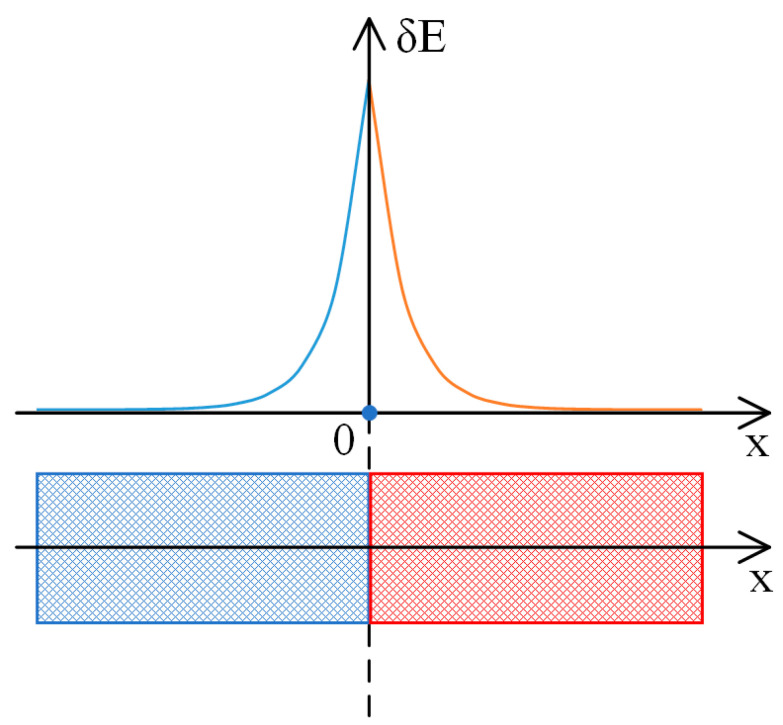
Behavior of solutions for the linearized Equation (35) in the neighborhood of the contact region between oppositely charged meshes.

The solution presented in Equation (46) indicates a decrease in value as one progresses leftward from the designated origin of the coordinates, which has been established at the intersection of the network with opposing charge signs. In contrast, the solution indicated in Equation (47) shows a decrease in value when moving in a rightward direction. The characteristics of these solutions are represented schematically in Figure 11. This example demonstrates that, for the chosen parameters, a double electric layer forms at the interface between oppositely charged networks. The size of these layers is much larger than that of the layers created at the solution–gel interface in equilibrium states. It should be recalled that for an electric field intensity of 10 V/m, the length of Λ is about 2.5 mm.

**Figure 12:**
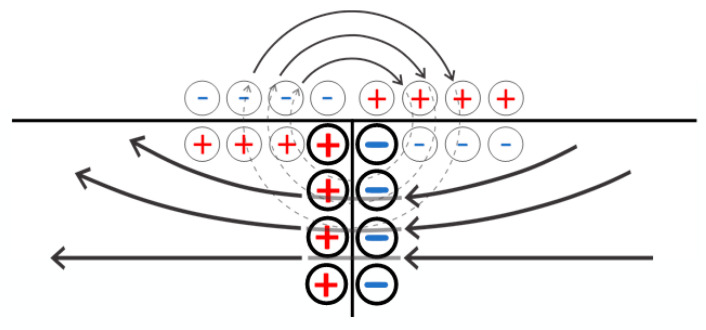
A simplified representation of the electric field structure in the contact zone of oppositely charged meshes during the flow of electric current. Bold circles indicate schematic charges corresponding to the electric double layer formed at the contact point of oppositely charged polyelectrolyte networks, while thin circles schematically represent the charges associated with the double layers formed at the network–solution interface.

The main difference is the amplitude of the double electric layers being formed at the solution–gel boundary and in the contact area of the oppositely charged meshes. Since the value of a positive electric charge generated within the negatively charged, left network in the contact zone is larger than the values of the charges which correspond to a layer formed at the gel and solution boundary, the structure of the field created by a double layer emerging due to the action of an external voltage source is topologically similar to the structure of the field of the usual flat capacitor (Figure 12). This means that close to the solution, near a contact point of oppositely charged meshes, there appears an electric field that forces negatively charged ions to move into the left network and positively charged ions to enter the right one (see Figure 10). The sign of these ions corresponds to the type of conductivity of the networks, which results in additional ion fluxes providing a depletion of the solution above the hydrogel.

This, increasing the amplitude of the double electric layer, provides the possibility to create an ’ion pump’ that can remove low-molecular-weight ions from the aqueous solution without having to pass an electric current through the solution itself. This effect can be used in the long term to develop novel measurement devices and modernize the existing techniques for demineralizing liquids containing low-molecular-weight salts.

## 6. Discussions

Thus, when a low-molecular-weight salt solution flows through a polyelectrolyte, an electromotive force (EMF) can arise. From a general standpoint (i.e., from the perspective of Onsager’s principle [56,57]), this effect is the counterpart to the effect that makes electrodialysis possible. However, with respect to polymer hydrogels, it has not been considered in the literature before, either from an experimental or a theoretical standpoint.

In terms of how it appears, this effect is close to the phenomenon of the flow potential—one of the most well-known electrophoretic phenomena [58,59]. For example, such a potential difference arises when a solution flows through a layer of sand, caused by the deformation of electric double layers at the interface between sand particles and the solution due to fluid motion. This is precisely what allows us to refer to the effect under consideration as an analog of the flow potential.

The phenomenon in question potentially has rather broad prospects for practical applications.

The most obvious field for the use of phenomena related to the spontaneous generation of electric fields during the flow of low-molecular-weight salt solutions through polyelectrolyte hydrogels concerns the empirical research of such gels. As a matter of fact, modern studies are more often making use of polyelectrolyte hydrogels that have a high level of complexity with respect to both composition and structural arrangement [60,61]. In addition to ionogenic functional groups, such materials can also contain hydrophobic groups and the species involved in selective interactions with specific low-molecular-weight compounds, and the like [62,63]. Heterogeneous hydrogels attract much attention from researchers too [64,65]. In this context, it is essential to note that the actual degree of the ionization of polyelectrolyte networks (the dissociation degree of functional groups) cannot be altered by any of the existing techniques [66]. There is no device to compare with existing pH meters that can measure the parameters of the medium in the polymer matrix without causing irreparable damage. The above-presented results unambiguously show that the electric fields caused by the motion or percolation of a low-molecular salt solution through polyelectrolyte networks are measurably determined by the mobility of the low-molecular ions in their structure.

The quantification of this parameter will also subsequently provide insights into the composition of polyelectrolyte hydrogels, among others. From the perspective of measurement methodology, a crucial distinction has to be realized. The direct measurement of the electrophysical properties of solutions is associated with a series of issues, including problems related to shielding and induced electric field generation. These concerns have been extensively documented in the classical literature on the subject [67,68]. The ability to quantify solution concentrations allows for the use of more reliable methods, such as those based on spectrophotometric principles. This at least allows for the comparison of results using different measurement methods. It is important to note that the use of a ‘flow’ potential difference for the examination of the electrophysical properties of polyelectrolyte networks has some peculiar advantages since it excludes the application of any external sources of power supply. In such systems, electromagnetic fields should appear naturally.

Concerning the theoretical models presented above, it now clearly appears that electrophysical and electrochemical parameter variations can satisfactorily be reduced by means of concentration measurements. It should be specified that this paper concentrates on the possible use of the results for measurement purposes. The theoretical implications of the results, however, transcend this aspect, with the potential development of new ways to demineralize solutions being just one example.

The traditional electrodialysis processes are at least theoretically predicated on the notion that an electric current facilitates the individual passage of negatively and positively charged ions in a depleted solution. Such a situation gives rise to a number of complications, including, among other things, problems related to the inherent properties of the formation of layers at the membrane interface. Of particular importance is the large increase in energy consumption that occurs when low-molecular-weight ions are removed from the depleted solution. The example illustrated in Figure 12 indicates how ion removal from a solution can occur when its ohmic resistance increases substantially owing to a decrease in ion concentration. Moreover, in this arrangement, the electric current flows through a medium whose ohmic resistance is low enough even when the concentration of ions in the original solution is small (for example, in accordance with sanitary norms). It is also worth emphasizing that the area in which electric fields that can act as an ‘electrophysical pump’ occur is very extensive, as follows from the above estimates of the parameter Λ. The size of the region exhibiting electrical inhomogeneity, being of the order of 2 mm, hints at the feasibility of a further miniaturization down to an analogue of a dialysis system with features of the order of 0.5 cm. It may be speculated that such a development could lead to an industrial-scale demineralization of fluids by a parallel coupling of such elements. Hence, our proposed mathematical model, being based on the linearization of the system of equations describing field distribution, allows us to assume that it is possible to work out such a variant of liquid demineralization by which many of the disadvantages of classical electrodialysis can be surmounted.

The authors understand that this conclusion is of crucial importance. Additionally, it can be profoundly confirmed only based on relevant experimental data. With the above consideration taken into account, at this stage of research, it seems more appropriate to develop the measurement techniques in such a way that the measurement of the electrophysical characteristics of the investigated systems—hydrogels—will be performed integratively with the concentration measurements. In this regard, it is important to note that advances in information technology substantially simplify the design and manufacturing of prototype measuring and laboratory equipment [69,70].

It is also important to point out that the methods based on the measurement of the electrophysical characteristics of polyelectrolyte grids can be used as a basement for the quite different levels of measuring devices in which the active substance is hydrogel. The simplest example is the concentration measurement of substances that are able to change the ionic composition of aqueous mediums in the air. Such devices are not required to undergo a complete demineralization of the solution formed by their interaction with water. It is enough to detect and measure the amplitude of the corresponding variations in concentration at the output of the above-mentioned systems.

## 7. Conclusions

Thus, when a low-molecular-weight salt solution flows through a polyelectrolyte hydrogel, an electromotive force is generated. Its polarity depends on both the outflow velocity of the solution and the sign of the network’s charge. This effect can be theoretically described by analyzing the charge balance in the system under consideration and by qualitatively examining the deformation of the electric double layers at the solution–gel interface.

This effect can be employed to develop experimental techniques for the study of the electrophysical characteristics of polymer networks.

In the long term, this effect can also be used to develop new methods for the demineralization of liquids. In this case, as follows from the proposed theoretical model, it is possible to divide the initial fluid flow into enriched and depleted streams. An advantage of these methods is that they reduce energy consumption by making use of gravitational forces, which ensure the percolation of the solution through a system of oppositely charged networks.

This method can also be further improved, as demonstrated by the additional mathematical models presented in this study.

## Data Availability

The original contributions presented in this study are included in the article. Further inquiries can be directed to the corresponding authors.
